# Common Cortical Loci Are Activated during Visuospatial Interpolation and Orientation Discrimination Judgements

**DOI:** 10.1371/journal.pone.0004585

**Published:** 2009-02-24

**Authors:** Marc S. Tibber, Elaine J. Anderson, Dean R. Melmoth, Geraint Rees, Michael J. Morgan

**Affiliations:** 1 Department of Optometry and Visual Science, City University, London, United Kingdom; 2 Institute of Cognitive Neuroscience, University College London, London, United Kingdom; 3 Wellcome Trust Centre for Neuroimaging, University College London, London, United Kingdom; Istituto di Neurofisiologia, Italy

## Abstract

There is a wealth of literature on the role of short-range interactions between low-level orientation-tuned filters in the perception of discontinuous contours. However, little is known about how spatial information is integrated across more distant regions of the visual field in the absence of explicit local orientation cues, a process referred to here as visuospatial interpolation (VSI). To examine the neural correlates of VSI high field functional magnetic resonance imaging was used to study brain activity while observers either judged the alignment of three Gabor patches by a process of interpolation or discriminated the local orientation of the individual patches. Relative to a fixation baseline the two tasks activated a largely over-lapping network of regions within the occipito-temporal, occipito-parietal and frontal cortices. Activated clusters specific to the orientation task (orientation>interpolation) included the caudal intraparietal sulcus, an area whose role in orientation encoding *per se* has been hotly disputed. Surprisingly, there were few task-specific activations associated with visuospatial interpolation (VSI>orientation) suggesting that largely common cortical loci were activated by the two experimental tasks. These data are consistent with previous studies that suggest higher level grouping processes -putatively involved in VSI- are automatically engaged when the spatial properties of a stimulus (e.g. size, orientation or relative position) are used to make a judgement.

## Introduction

Since Hubel and Wiesel [1] discovered that individual cells in the occipital cortex are sensitive to the onset of individual bright or dark bars with a specific orientation much progress has been made in the characterisation of receptive field (RF) anatomy, circuitry and physiology. (See [2] for review). However, some 40 years later, it is still not clear how contour segments in disparate regions of the visual field are perceptually integrated. This ‘linking’ of information is a critical intermediate stage in the perception of visual form [3] and has lead to the notion of an ‘association field’ [4], [5] that integrates information from neighbouring filters tuned to similar orientations (see Field and Hayes [6] and Hess and Field [7] for reviews). However, as well as being able to ‘bridge the gaps’ within discontinuous contours, the human visual system is capable of interpolating a path or trajectory in the absence of explicit local orientation cues, a process referred to here as visuospatial interpolation (VSI) [8], [9].

**Figure 1:**
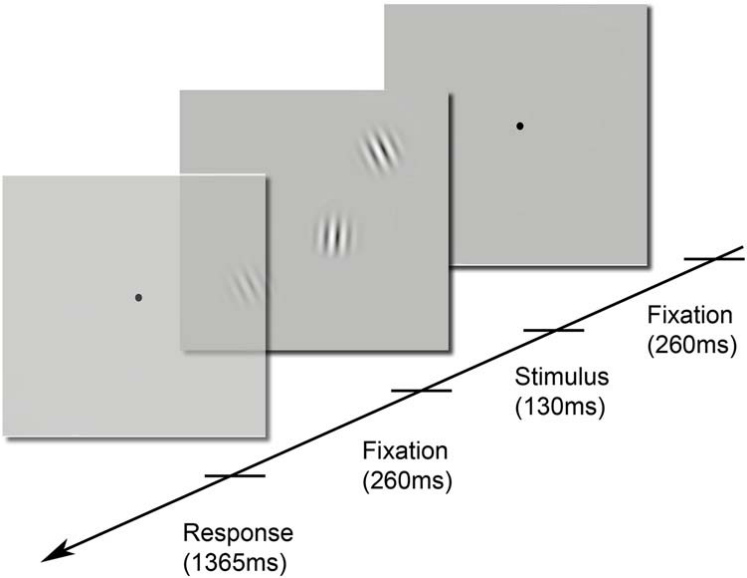
Stimulus presentation sequence. Each trial lasted 2015 ms and consisted of: a central fixation (260 ms) period, followed by the presentation of the stimulus (130 ms) and a second fixation period (260 ms) before a response period (1365 ms) in which the observers (Os) had to give a left or right key press response. Os fixated centrally throughout each trial, although the central fixation point temporally disappeared with the appearance of the stimulus. The stimulus consisted of three Gabor patches (3 cpd, σ = 0.43): two flanking patches forming a 45° reference line and a central target patch. Time flows from right to left. Interpolation task: Os judged whether the central target was offset above and to the left or below and to the right of the 45° reference line. Orientation task: Os judged whether the carrier grating of the central target patch was oriented clockwise or anticlockwise relative to the carrier orientation of the flankers. Sensorimotor control task: Os maintained fixation during stimulus presentation and alternately pressed left and right keys on successive trials.

As an example of VSI, the collinearity of dots or Gaussian blobs (e.g. vernier acuity) can be judged with some degree of accuracy, even with a large separation of the stimulus elements (Figure 1, middle frame). The fact that performance with widely separated targets is relatively unimpaired when patches of different spatial frequency [10], orientation [11], colour [11] or contrast polarity [12], [13: Fig. 4.12] are used, or when irrelevant ‘distracters’ are placed between the targets [9] suggests that simple linear filters alone are not capable of carrying out the computation. Unless one assumes that relative position is implicitly encoded in patterns of activation within early topographic maps [3] one must hypothesise the existence of a second stage to the computation that either integrates information from low level filters [14], [15], [9], [16], [13] or endogenously generates ‘virtual contours’ between the stimulus elements [3], [16]. Candidate regions for this second stage to the process of VSI are therefore the posterior parietal cortex, which has been implicated in spatial processing [17]–[21] and visual feature binding [22], and the occipito-temporal cortex, which is thought to underlie global integration of local signals [23]–[25] and the perception of subjective / virtual contours [26]–[29].

To examine higher level correlates of VSI, functional magnetic resonance imaging (fMRI) was used to measure brain activity whilst human observers performed a visuospatial task involving interpolation across space: a three-element alignment task [8], [10], [30] in which judgements of collinearity were made using Gabor patches (see Figure 1). Brain activation evoked by such VSI (relative to a low-level fixation baseline) was compared with that evoked by two other blocked tasks that used identical stimuli: observers performed a simultaneous orientation discrimination (a spatial task that lacks an interpolation component; [31]), and a sensorimotor control task in which observers passively viewed the stimulus and alternately pressed left and right keys on successive trials. Analysis was focused on the occipito-parietal and occipito-temporal cortices, areas associated with spatial processing and local feature integration.

## Results

**Figure 2:**
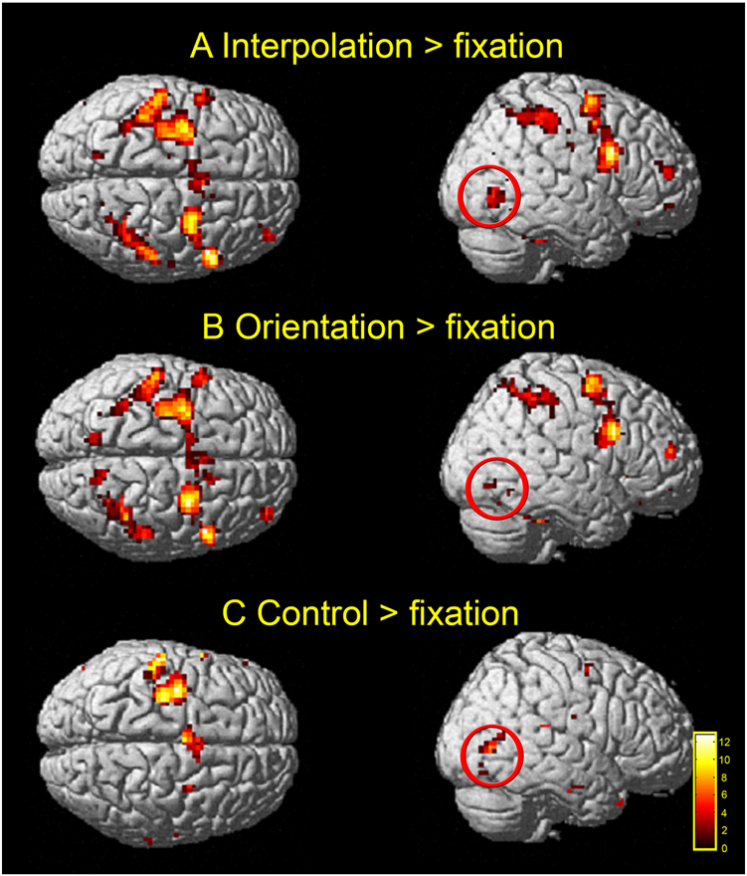
Experimental conditions compared to fixation. Clusters activated (relative to fixation) from the random effects group analysis [P<0.001 (uncorrected); see Table 1 for activated foci] are shown for each condition: (A) interpolation, (B) orientation and (C) sensorimotor control on rendered images of a standard brain [Montreal Neurological Institute (MNI)]. Note the lack of posterior parietal activity in the sensorimotor control condition (C) compared to the extensive activations in the two task conditions (A&B), which is focused along (though not restricted to) the intraparietal sulcus. Ringed in red is a region of the occipito-temporal cortex (possibly the lateral occipital complex) that is present in all three conditions, and though only shown in one hemisphere here, was actually activated bilaterally (see Table 1).

**Figure 3:**
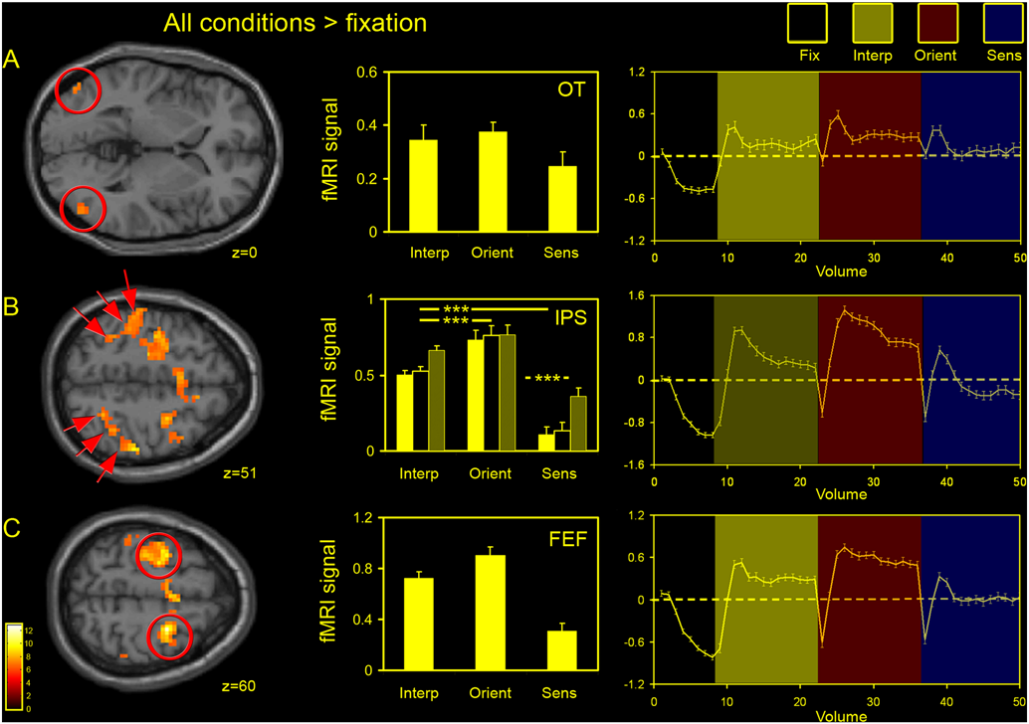
Experimental conditions compared to fixation. Clusters activated (relative to fixation) in the random effects group analysis are shown here for all conditions [left column; threshold of P<0.001 (uncorrected); see Table 1 for activated foci] along with plots of % BOLD signal change relative to fixation (centre column; group data) and the corresponding averaged time-series (right column) for the following key activated regions: (A) an occipito-temporal (OT) region, (B) the intraparietal sulcus (IPS), and (C) frontal eye fields (FEFs). Plots represent activity combined for each cluster across the two hemispheres relative to fixation. For the intraparietal sulcus (B) clusters of activity were divided into three groups bilaterally, generating three sub-regions of interest (red arrows) moving from posterior regions of the IPS towards more anterior regions. In the plot the posterior region is shown in bright yellow, the middle region in black and the more anterior region in light yellow / grey. The z coordinate (MNI) is given for each horizontal slice shown. Conditions: [Inter, interpolation; Orient, orientation; Sens, sensorimotor control]. *** *P*<0.001 (one-way ANOVA with Tukey's *post hoc* analysis).

**Figure 4:**
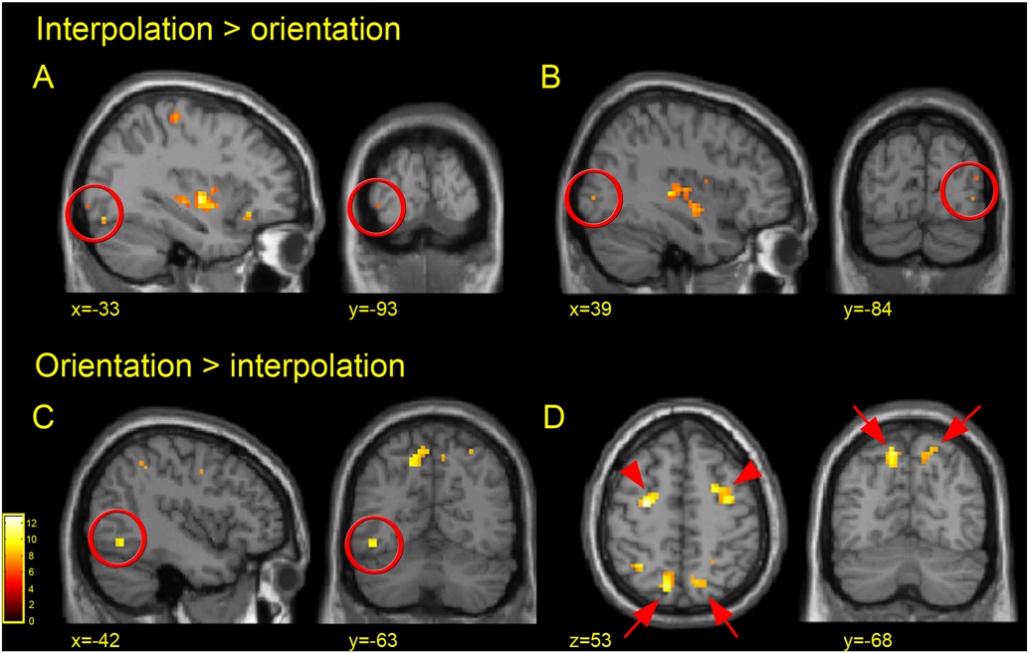
Task-specific activations. Activated clusters for the two critical contrasts: (A&B) interpolation versus orientation and (C&D) orientation versus interpolation are shown from the random effects group analysis [P<0.001 (uncorrected); see Table 2 for full list of activated foci]. In (A) small activated clusters (1–2 voxels in size) are seen in the left fusiform gyrus and left inferior occipital gyrus. In (B) activations are seen in the right inferior and right middle occipital gyri. However, the middle occipital gyrus cluster (upper cluster) is not due to activity in the interpolation condition but a de-activation in the orientation condition relative to fixation. (See text and Figure 5 for information on task-specific deactivations). In the orientation versus interpolation contrast activations are seen in an anterior region of the left fusiform gyrus (C), and bilaterally in the frontal eyefields [red arrow-heads (D)] and caudal regions of the intraparietal sulcus [red arrows (D)]. MNI coordinates are given for each slice presented.

**Figure 5:**
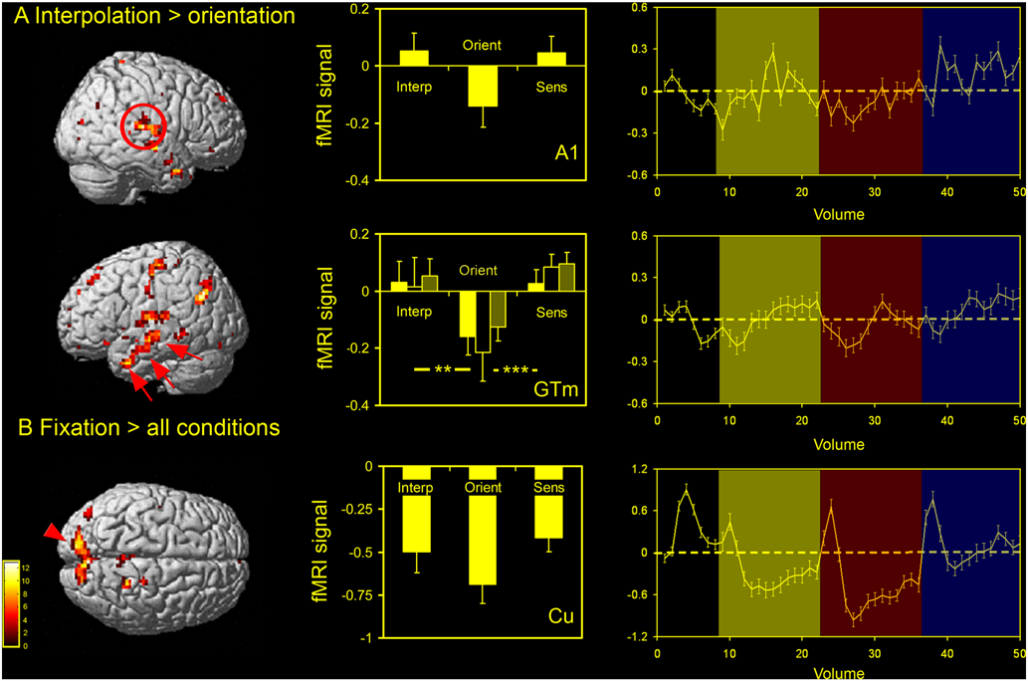
Deactivations relative to fixation. Clusters of relative activation / deactivation from the random effects group analysis [see Table 2 for foci; P<0.001 (uncorrected) threshold] are shown along with plots of % BOLD signal change relative to fixation (centre column; group data) and the corresponding averaged time-series (right column) from the following contrasts: (A) interpolation versus orientation and (B) fixation versus all conditions. Several clusters of relative activity in the interpolation versus orientation contrast were found to be due to de-activations in the orientation condition (% BOLD signal change relative to fixation). Significant clusters include the primary auditory cortex bilaterally (A1: red ring) and the middle temporal gyrus bilaterally (GTm: red arrows). For the GTm, clusters of activity were divided into three groups bilaterally, generating three sub-regions moving from posterior, through central and on towards more anterior regions (yellow, black and light yellow / grey plots respectively). The only region showing deactivations (relative to fixation) in all conditions was a large midline cluster pertaining to the pre-cuneus / cuneus (Cu: red arrow-head), a well-documented component of the ‘default network’. (See text for details). Conditions: [Interp, interpolation; Orient, orientation; Sens, sensorimotor control]. ** *P*<0.01; *** *P*<0.001 (one-way ANOVA with Tukey's *post hoc* analysis).

Anatomical regions are defined in MNI coordinates (following normalisation of each participant's data to the ICBM-152 template), which closely approximates to the space described by Talairach and Tournoux [32]. Where coordinates are given for clusters of activity common to two or more tasks they denote those taken from the interpolation versus fixation contrast. If coordinates from previous studies are provided, the letters ‘MNI’ or ‘TAL’ (appearing in parenthesis) indicate whether they are in MNI or Talairach space respectively. Details from all contrasts are provided in the accompanying tables (Tables 1 & 2) and figures; note that in the figures task-specific / common activations (Figures 2, 3 and 4) are presented separately from de-activations (Figure 5).

**Table 1:** Experimental conditions versus fixation.

Region			Interp.>fixation	Orient.>fixation	Sens.>fixation
			x y z (Z)	x y z (Z)	x y z (Z)
**Parietal**	**PPC**	**L**	−42 −39 39 (4.3)	−36 −48 51 (3.5)	ns
		**R**	30 −51 48 (4.3)	27 −42 45 (4.4)	ns
	**GPoC**	**L**	−51 −27 42 (4.4)	−51 −27 45 (4.1)	−48 −24 39 (4.3)
		R	39 −33 45 (3.4)	48 −30 39 (3.2)	ns
	**PO Su**	**L**	−27 −69 21 (3.3)	ns	ns
		**R**	30 −66 27 (3.6)	30 −63 30 (3.9)	ns
**Temporal**	**LO**	**L**	−48 −78 0 (3.3)	−45 −78 −6 (3.5)	−45 −78 3 (3.6)
		**R**	42 −63 −3 (3.7)	48 −75 −9 (3.2)	48 −69 3 (3.7)
	**LO_a_**	**R**	45 −57 −12 (3.2)	42 −57 −12 (3.8)	ns
**Frontal**	**M1**	**L**	−33 −18 63 (4.1)	−30 −15 57 (4.1)	−33 −21 60 (3.9)
	**FEF**	**L**	−36 −6 57 (4.4)	−33 0 63 (4.5)	−42 −9 57 (4.4)
		**R**	27 −3 60 (4.5)	30 −3 57 (4.6)	30 −3 57 (3.7)
	**SEF**	**M**	0 6 57 (4)	−3 9 51 (3.9)	0 6 54 (4.3)

The MNI coordinates and Z scores of peak activity are shown for activated clusters from the group random effects analysis (see Methods) in the parietal, temporal and frontal lobes along with the putative anatomical / functional regions involved. A threshold of P<0.001 (uncorrected) is used in accordance with our prior experimental hypotheses (see Introduction). PPC, posterior parietal cortex; GPoC, post-central gyrus; PO Su, parieto-occipital sulcus; LO, dorsal caudal subdivision of the lateral occipital complex (LOC); LO_a_, anterior ventral projection of the LOC; M1, primary motor area; FEF, frontal eyefield; SEF, supplementary eyefield; L, left hemisphere; R, right hemisphere; Interp., interpolation; Orient., orientation discrimination; Sens, sensorimotor control.

**Table 2:** Direct comparisons of experimental conditions.

Region			Interpolation>orientation	Orientation>interpolation
			x y z (Z)	x y z (Z)
**Parietal**	**PPC**	**L**	ns	−30 −54 54 (3.8)
		**R**	ns	24 −51 54 (3.15)
	**h cIPS**	**L**	ns	−12 −69 54 (4.03)
		**R**	ns	12 −66 54 (3.5)
	**PostCG**	**L**	−48 −15 57 (4)	ns
Temporal	**FFG**	**L**	−33 −81 −15 (3.8)	−42 −63 −12 (3.8)
	**InfOG**	**L**	−33 −93 −6 (3.1)	ns
		**R**	39 −84 0 (3.4)	ns
Frontal	**FEF**	**L**	ns	−27 −9 54 (4.4)
		**R**	ns	36 −3 57 (3.9)

The MNI coordinates and Z scores of peak activation are shown for critical clusters in the parietal, temporal and frontal lobes from the group random effects analysis (see Methods) along with the putative anatomical / functional regions involved. A threshold of P<0.001 (uncorrected) is used in accordance with our prior experimental hypotheses (see Introduction). h cIPS, human caudal intraparietal sulcus; PPC, posterior parietal cortex; PostCG, post-central gyrus; FFG, fusiform gyrus; infOG, inferior occipital gyrus; FEF, frontal eyefield; L, left hemisphere; R, right hemisphere.

### Psychophysical performance during scanning

Observers were clearly able to perform the tasks successfully inside the scanner: the average performance was 82.4%, only marginally less than the prediction based on offline performance (84%). As the data were found to be normally distributed (all *P*s>0.9; one-sample KS test) performance on the two tasks were compared in an independent samples t-test. Performance in the interpolation (83±5.7%) and orientation (81.7±4.6%) tasks did not differ significantly (t_(16)_ = 0.5, *P* = 0.63). Thus, task difficulty was matched between the two main conditions.

### Fixation analysis

To determine whether observers were able to maintain fixation throughout the experiment gaze direction was monitored using an infrared video-based eye tracker. Reliable eye position data were only available for 6 out of the 9 observers scanned. This was due to technical difficulties in tracking the corneal reflection when corrective lenses were used in the scanner and an equipment failure during one session. For the data gathered both the amplitude and frequency of saccades were found to be normally distributed (*P*>0.8 and *P*>0.5 respectively; one-sample KS test). A factorial ANOVA of saccade amplitude with condition as one factor (3 levels: interpolation, orientation and sensorimotor control) and saccade direction as a second factor (2 levels: horizontal and vertical) showed no significant effects of condition (F_(2,30)_ = 1.14, *P*>0.33) or saccade direction (F_(1,30)_ = 0.21, *P*>0.65). A similar analysis of saccade frequency failed to show any effects of condition (F_(2,30)_ = 1.63, *P*>0.21) or direction (F_(1,30)_ = 0.84, *P*>0.77). Thus, fixation accuracy was the same for the two main tasks and the sensorimotor control task.

### Task conditions versus fixation (Table 1)

Areas activated for the comparison of each of the experimental conditions (versus fixation) for the group random effects analysis are listed in Table 1; corresponding statistical parametric maps overlaid on anatomical brain images are shown in Figure 2. In addition, the % BOLD signal change (relative to fixation) is presented for selected activated clusters along with time-locked average responses (Figure 3). Eyes-open fixation represents the most suitable baseline with which to compare the 3 experimental conditions, as the oxygen extraction fraction (OEF) is relatively uniform across the brain in this state [33]. It is worth mentioning that whilst activity was not identified in primary visual areas at the group level (Figure 3), this is likely to reflect the fact that: (1) the stimuli used were relatively small, spatially jittered and presented for a brief duration (130 ms), and (2) the data presented have been stereotactically normalised and averaged across multiple observers.

In the parietal cortex a large swathe of bilateral activity was evident along the intraparietal sulcus (IPS); this was specific to the two tasks of interest – VSI and orientation discrimination. In contrast, this pattern of activity was not seen in the sensorimotor control condition (Figure 2 and Figure 3B). Elsewhere in the parietal cortex there was bilateral activation of the post-central gyrus (GPoC), which most probably corresponds to the primary somatosensory cortex (S1), as well as in the occipitoparietal sulcus (PO Su). Bilateral activity in these areas (relative to fixation) was also limited to the orientation and interpolation tasks - with the exception of the occipitoparietal sulcus in which activity was lateralised to the right hemisphere for the orientation task. In fact, parietal activity in the sensorimotor control condition was restricted to the left somatosensory cortex, which is probably associated with contralateral button presses. Thus, the parietal cortex (other than motor association areas) appears only to have been engaged during performance of an active task. These may represent general effects of attention or specific computations associated with the task, or indeed a combination of both. Finally, one site in the occipital / parietal cortex -the cuneus / precuneus (−15 −90 30; 6 −78 33) - was deactivated in all conditions relative to fixation. This corresponds to a well documented component of the default network [33]–[35]. See Figure 5B.

Task-related activation was also found in two distinct areas in a portion of the occipito-temporal cortex. These locations are similar to those associated with previous reports of sub-divisions of the lateral occipital complex (LOC), an area in the ventral stream sensitive to form and shape that is likely to support the process of object recognition [36]. See Figure 3A. These areas may represent the dorsal caudal subdivision (LO: −48 −78 0; 51 −72 −3) and a more anterior ventral projection (LO_a_: 45 −57 −12). The corresponding coordinates cited by Grill-Spector and colleagues [37] are: −41±5 mm, −77±6 mm and 3±7 mm (x-, y- and z- coordinates; TAL) and −38±5 mm, −50±6 mm and −17±5 mm for the left LO and LO_a_ respectively. It is worth noting however that activity in the caudal region reported here (the putative area LO) also overlaps with previous reports of the human motion processing complex h MT+/V5 [38].

In the frontal cortex we observed activation (relative to fixation) associated with the interpolation and orientation tasks and the sensorimotor control condition in the left pre-central gyrus, possibly associated with motor activity in the primary motor cortex (M1) related to the participantś button presses. In addition, there was activity in regions that correspond to previous reports of the frontal eyefields (FEFs: [39], [40]) and supplementary eyefields (SEF: [41]), again evident in all three conditions (Figure 3C).

### Direct comparisons between interpolation and orientation tasks

The contrasts of interpolation versus orientation and orientation versus interpolation highlighted a number of task-specific activations (Figure 4; Table 2), as well as extensive task-specific de-activations (Figure 5A). These could be distinguished by plotting the % BOLD signal change (relative to fixation) for each condition, and will be discussed separately. Considering the contrast of interpolation versus orientation first (Figure 4A & 4B), no voxels in the PPC were significantly activated. The only area of activation in the entire parietal cortex was in fact restricted to the post-central gyrus (PostCG) of the left hemisphere, probably corresponding to the somatosensory cortex. Exactly why the two tasks differ with respect to activity in this area is unclear, as the number of button presses was identical in the two tasks. In the occipito-temporal regions of the ventral stream, activations were limited to several small foci of activity (1–2 voxels in size) in the inferior occipital gyrus (infOG) and fusiform gyrus (FG). Consequently, outside of the somatosensory cortex, there is little evidence for localised activity specific to the VSI condition.

In addition to these limited task-specific activations associated with the process of VSI, there were many clusters to emerge from the contrast of interpolation versus orientation that represented deactivations (relative to fixation) in the orientation condition. Recent studies have highlighted cortical deactivations as genuine phenomena that may reflect reductions in neuronal activity [42], [43]. To visualise these, levels of activity relative to fixation were plotted from several activated clusters defined by the contrast of interpolation versus orientation (Figure 5A). Areas deactivated in the orientation condition (relative to fixation) included the angular gyrus (−39 −63 30; 54 −66 30), a cluster in the left parietal cortex (−24 −36 57), the right mid occipital gyrus (45 −81 15) as well as extensive areas of the temporal cortex that ran from the posterior regions of the middle temporal gyrus (−45 −18 −6; 66 −12 −6) to the anterior (−45 6 −33; 54 3 −33). In addition, deactivation was found bilaterally in the primary auditory cortex as defined anatomically by Heschl's gyrus (−40 −22 10; 40 −22 11). This is in agreement with previous studies that have shown auditory and / or middle temporal gyrus deactivations during tasks that involve visual imagery [44], passive visual stimulation [45], visual attention [46], face and location matching [47] and perhaps most interestingly, during a study of perceptual discriminations based on orientation and size [20].

In the contrast of orientation versus interpolation (Figure 4C & 4D), a very different pattern emerged. There were two bilateral foci of differential activation in the parietal cortex. The first pair was more posterior and medial (Figure 4D red arrows) and most probably relates to the human caudal intraparietal sulcus (h cIPS). Regions in the human cIPS and posterior IPS have previously been implicated in the visual processing of orientation in reaching and grasping studies [19], as well as several purely perceptual experiments involving visual priming to object rotations ([48]: −13 −72 38; 25 −74 35; TAL), surface orientation discriminations on the basis of texture gradients ([49]: −16 −68 57; 20 −67 58; MNI), object orientation matching ([19]: 34 −52 44; TAL), object rotation discriminations of 2D and 3D shapes ([20]: 20 −64 58; 20 −70 50; TAL), as well as grating orientation discriminations ([50]: −16 −72 52; 16 −68 60; TAL / [51]: −24 −58 48; 22 −58 48; TAL). Thus, it would seem that even for a purely perceptual task, orientation processing invokes activity within the dorsal stream / PPC, and more specifically, within the posterior regions of the IPS. Furthermore, a study showing that a region within the IPS is activated both by visual and tactile orientation tasks ([52]:30 −54 56; MNI) suggests that regions of the PPC may underlie multisensory processing of orientation.

In contrast, activity in the ventral stream was limited to a single site in the left fusiform gyrus (Figure 4C). The fusiform / inferior temporal gyri have previously been implicated in orientation discriminations, although these usually tend to involve bilateral activity ([50]:−52 −60 −12; 48 −64 −12; TAL / [51]: −28 −76 −16; 38 −70 −16; TAL / [53]: −50 −68 −16; 56 −58 −14; TAL) or lateralisation to the right hemisphere ([31]: 40 −62 −12; TAL / [54]: 40 −62 12; TAL). One possible reason why activity in this condition was lateralised to the left hemisphere is that it may have reflected a need to attend to the local orientation of the Gabor patches in the orientation condition as opposed to the global orientation defined by their relative position [55], [56]. Additional activation was seen bilaterally in the FEFs. Since the tasks did not differ with respect to performance levels or patterns of fixation it is unlikely that this is a general effect of attention or eye movements.

## Discussion

This study was designed to examine the neural correlates of VSI, and more generally, to shed light on the mechanisms involved in the encoding of relative position. Observers used identical stimuli to perform a three-element alignment task [8], [10], [30] that involved a process of spatial interpolation and an orientation discrimination task that required no explicit processing of target position. Relative to fixation both tasks activated an extensive network of areas within occipito-temporal, occipito-parietal and frontal cortices, reflecting a high degree of commonality between the two. This was to be expected: both conditions required observers to maintain fixation, view identical coherent stimuli, distribute their attention across multiple sub-regions of the stimulus, make a decision and map sensory input on to a motor response. In contrast, activation differences between the two conditions should reflect the unique and specific demands of the tasks, as patterns of fixation, spatial attention and task difficulty were controlled for.

Activations specific to the orientation discrimination task were seen in areas including caudal regions of the IPS (cIPS), a putative homologue of the monkey cIPS [57], [58], which contains neurons selective to binocular disparity [59] and orientation under a range of different conditions. (See Sakata et al. [60] for a review). The cIPS/PPC has been implicated in mental rotation [61], [62], the processing of object orientation during reaching and grasping [19] and orientation discrimination / classification of 2D / 3D shapes, graspable objects and surfaces [19], [20], [49], [48], [63]–[65]. In an attempt to accommodate these findings within the duplex model of vision, which maps vision for action and vision for perception on to the dorsal and ventral streams respectively [66]–[68] it has occasionally been claimed that the PPC (a dorsal stream area) is sensitive only to the orientation of graspable objects [48], [69], [64]. However, the data reported here do not support this view; instead, they contribute to a growing body of evidence that suggests the PPC is involved in orientation processing independent of stimulus type [51], [50], [52], [70] and may even integrate orientation information from multiple sensory modalities [52].

Surprisingly, once deactivations in the orientation condition were accounted for (relative to fixation), there was little evidence for task-specific activations associated with the process of VSI (VSI>orientation). However, Altmann et al. [71] provide comparable evidence to suggest that whilst common regions in the ventro-temporal and lateral occipital regions are involved in both object identification and orientation classification, stronger responses were consistently associated with the orientation task. In contrast, not a single cortical site showed elevated activation during the object identification task [71]. These findings are of particular relevance to the data reported here as object identification is a high-level task that is likely to include VSI as an early grouping process [72]. However, as the authors [71] go on to discuss, the absence of task-specific activations in a direct contrast between closely matched tasks of this kind may be interpreted two different ways: firstly, the same cortical areas may undertake the computations involved in both judgements. Hence, no distinct region will be highlighted in a direct contrast between the two. Alternatively, it is possible that both tasks are automatically performed when the observer is presented with an object, or in the case of our own stimuli, a coherent stimulus that may be integrated to form a Gestalt [73]. In this way, distinct cortical networks may in fact underlie the two tasks, whilst still remaining hidden in direct contrasts between them.

There is considerable evidence to suggest that when observers attend to the spatial properties of a stimulus (e.g. orientation, size or shape), higher level (semantic / global) processing of the image is engaged automatically [74]–[78]. Thus, in a series of perceptual matching tasks using line drawings of objects and animals, Boucart and colleagues have shown that when matches are made on the basis of the spatial properties of the stimulus there is a semantic interference effect. For example, response times on orientation judgements are either facilitated or inhibited depending on whether the reference or distracter are semantically related to the target, even though this information is irrelevant to the task [75], [78]. Further, the effect is seen irrespective of whether the orientation judgement is based on the principal axis of the stimulus (judgement of global form), or on a shorter line segment within the stimulus (judgement of local form; [77], [78]). In contrast, this interference effect is eradicated if judgements are made on overall stimulus colour or luminance (i.e. basic surface properties; [75]). Further, they have shown that areas within the occipitotemporal cortex, which are associated with perceptual grouping, shape analysis and semantic processing, are activated during an orientation classification task [79], [80], [53].

Automatic engagement of high-level ventral stream areas during spatial judgements of a stimulus is consistent with the data reported here; thus, extensive occipitotemporal activations were found in both experimental conditions. In fact, robust occipitotemporal activations were even reported when observers passively viewed the stimulus (sensorimotor control condition). However, in the absence of attentional engagement it is possible that observers performed one of the other tasks (orientation discrimination or VSI) during this condition, despite specific instructions not to do so. Further, as parietal areas were also activated in the two experimental conditions we are unable to rule out the possibility that dorsal stream areas may also be involved in the process of VSI. However, taken together, these data and observations suggest that the absence of task-specific activations in the VSI task (VSI>orientation) may reflect the fact that when observers are encouraged to make judgements of local orientation only, cortical regions associated with the perceptual organization of local stimulus elements into global shapes (in which VSI plays a role; [23]–[25]) are automatically engaged [79], [53], [71].

In conclusion, we have shown that judgements of relative orientation and stimulus collinearity activate a similar / over-lapping network of brain regions that incorporate both dorsal and ventral stream areas. In addition, our results provide only limited evidence for any specific brain regions activated during VSI over and above those regions implicated in judgements of stimulus orientation. Although evidence from previous studies are consistent with an interpretation of these data in terms of an automatic engagement of global grouping processes in both experimental conditions future studies are needed to rule out the possibility that task-specific activations were not seen during VSI because the two sets of computations involved were supported by distinct cell populations that lay beyond the spatial resolution of the imaging technique employed. To begin to tease apart these two hypotheses more sophisticated experimental paradigms such as fMRI-adaptation [81] and / or dual interference tasks will have to be employed. In parallel, the use of transcranial magnetic stimulation may be useful in determining which cortical activations are critical, and which merely incidental, to the process of visuospatial interpolation [82].

## Materials and Methods

Nine volunteers aged between 19 and 37 years (6 male, 3 female) with normal or corrected to normal visual acuity took part in the study. Seven were right-handed and 2 were left-handed according to the Edinburgh handedness inventory. Each gave informed written consent to participate in accordance with the Helsinki Convention and National Institutes of Health (NIH) guidelines for human subject experiments. The experiment was approved by the Institute of Neurology and National Hospital for Neurology and Neurosurgery Joint Ethics Committees.

### Stimuli

All stimuli were generated in MATLAB (The MathWorks, Natick, MA) using the Psychophysics Toolbox [9], [83] and projected onto a gamma-corrected backlit projection screen (spatial resolution 800×600, temporal resolution 60 Hz). Observers lay supine in the MRI scanner and viewed stimuli at 61 cm via an angled mirror mounted on the head coil. The stimulus consisted of three Gaussian-windowed sinusoidal gratings (Gabor patches) presented at a Michelson contrast of 72% and a mean luminance equal to the background grey (10 cd/m^2^). At the correct viewing distance, these patches had a spatial frequency (s.f.) of 3 cycles per degree (cpd); the standard deviation of the Gaussian envelope was 0.43°.

The centroids of the two outer patches were always positioned 5.1° from fixation, with one in the upper right quadrant and the other in the lower left, thus forming a reference axis that fell 45° to vertical. The central target was presented about fixation. This design was used instead of a vertical or horizontal alignment in order to minimise the availability of an internal cardinal axis representation as a cue to the tasks. The carrier gratings of the two outer (flanking) patches were always iso-oriented and presented randomly from trial to trial at 10°, −10°, 20° or −20° relative to vertical. The orientation of the flanker gratings was thus always incongruent with the orientation of the reference axis. The relative position and orientation of the central target patch was manipulated for each observer according to their individual thresholds (see below for details). In addition, the position of the whole stimulus (two flanking patches and one target) was randomly jittered as a rigid structure in the x- and y- planes on each trial by anywhere up to 0.36°, above and to the left, or below and to the right of the fixation point. The extent of this jitter was determined from pilot studies to be sufficient to abolish the relative position of the fixation point as a reliable cue to perform the interpolation task.

### Tasks and experimental design

In a block design paradigm observers performed one of three tasks:

1. A three-element alignment task [10], [30] (interpolation condition) using three Gabor patches - see Figure 1, middle frame. Observers judged whether the central target was offset above and to the left or below and to the right of an implicit line connecting the centroids of the two flankers (outer patches). The two offsets were presented in a pseudo-random sequence with equal probability.
2. A simultaneous orientation discrimination task. Observers had to judge whether the carrier grating of the central target patch was oriented clockwise (CW) or anticlockwise (ACW) relative to the carrier orientation of the flankers. The two orientations were presented in a pseudo-random sequence with equal probability.
3. A sensorimotor control task. Observers had to maintain fixation during stimulus presentation and alternately pressed left and right keys on successive trials.

Identical stimuli were used for all three experimental conditions: a task irrelevant spatial offset was presented in the orientation discrimination condition, and similarly, an irrelevant orientation cue was presented in the interpolation condition. See Figure 1 for details on the time-course of each trial. Note that on each trial (duration 2015 ms) the visual stimulus was presented only very briefly (130 ms), thus reducing the likelihood of eye movements. Observers fixated centrally throughout and responded with a key press within a 1365 ms time window after the stimulus had been extinguished. Responses were given by right-hand key presses with the exception of one participant who used his left hand.

A block design paradigm was used in which blocks of the experimental conditions (interpolation, orientation and sensorimotor control) were interleaved with blocks of a low-level fixation baseline - a central fixation spot presented against a background grey of 10 cd/m^2^. Each block consisted of 16 trials.

Across all three conditions the stimulus structure was identical - only the task instructions differed; these were cued at the end of each fixation block by a short text string presented centrally at fixation. To ensure that levels of attention were matched across the two experimental tasks the target offset and rotation relative to the flankers was defined by each individual's thresholds, which were measured offline using standard psychophysical procedures (see below).

### Psychophysical measurement of thresholds

Individual interpolation and orientation thresholds were measured outside the scanner using a setup that matched conditions inside the MR scanner as closely as possible (800×600 images presented at 60 Hz on a gamma corrected display). The only differences were that outside the scanner stimuli were presented on a CRT monitor and the average luminance of the screen was set to 13 cd/m^2^ (as opposed to 10 cd/m^2^). In addition, in contrast to the fMRI experiment the response period was not of a fixed duration: instead, observers set the pace at which sequential trials were presented facilitating a relaxed experimental setting. [Note: prior to scanning sessions individual observers also practiced the tasks using the precise sequence timings used inside the scanner. In these practice sessions the restricted response times used (1365 ms) were found to be of sufficient duration for the observers to undertake the tasks comfortably].

As the orientation of the Gabor patches did not affect interpolation thresholds, but conversely, orientation thresholds varied as a function of target offset (as determined from pilot data), interpolation thresholds were measured first using iso-oriented target and flankers. Orientation discrimination thresholds were then measured in a separate block with the target patch offset by an amount equal to the individual's interpolation threshold. The absolute (unsigned) target envelope offset or carrier orientation, depending on the procedure, were adjusted between sequential trials using a 2 down 1 up staircase procedure. The sign of the offset / orientation cue was randomly determined on each trial. Each experimental run finished after 200 trials were completed. No feedback with respect to performance was given. The first run was considered a practice session, and the last 2 runs were used for the analysis. Data were fitted with a cumulative normal function and the threshold calculated as the mean of the 2 values of sigma obtained (1 per run). This corresponded to a performance level of 84%. Finally, observers practiced the precise sequence to be used in the scanner to ensure that they were comfortable with the procedure.

### Scanning details

A 3T Siemens Allegra head scanner with standard head coil was used to acquire all functional and structural data. A standard high resolution EPI sequence (matrix 128×128, field of view 192 mm, in-plane resolution 3×3 mm, slice thickness 2 mm with a 1 mm gap, TE 65 ms, TR 2340 ms) was used to acquire 36 slices positioned to optimise coverage of the occipital and parietal lobes. High resolution T1-weighted structural images (1×1×1 mm) were also acquired.

Observers performed 6 functional scan runs (7.72 minutes each) in a single scanning session. Each run comprised of experimental blocks (16 trials of a single condition) lasting 32.76 sec each (14 volumes) interleaved with 18.7 seconds of fixation (8 volumes). Each of the 3 experimental conditions (interpolation, orientation and sensorimotor control) was presented twice in a single run in a counterbalanced order. The order in which the experimental conditions were presented was reordered between scan runs and between observers. An additional 5 volumes (11.7 seconds) were acquired at the start of each scan run to ensure that the brain had reached steady state magnetisation.

### fMRI data analysis

Image processing and statistical analyses were carried out using SPM5 (The Wellcome Trust Centre for Neuroimaging, UCL, http://www.fil.ion.ucl.ac.uk/spm). The first 5 images from each experimental run were discarded. The remaining images were realigned to the first image to compensate for head movements, spatially normalised to an EPI template provided with SPM-5 [the ICBM-152, as defined by the Montreal Neurological Institute (MNI)], and spatially smoothed with an isotropic smoothing kernel (7 mm full width at half maximum). A linear combination of regressors representing the time series for each of the 3 experimental conditions (interpolation, orientation and sensorimotor control) and fixation baseline (fixation) were convolved with a synthetic haemodynamic response function and its temporal derivative, creating a box car function. The general linear model (GLM) was then used to generate parameter estimates of activity at each voxel, for each condition. Linear contrasts between regressors, representing the different experimental conditions, were used to determine activated brain areas by generating statistical parametric maps of the t-statistic [SPM(t)].

First level (observer-specific) contrasts were constructed for each of the experimental conditions (interpolation, orientation and sensorimotor control) compared to fixation baseline (fixation). For each contrast, a single image was generated, carrying information about cortical areas engaged during that task relative to baseline. Further contrasts were made comparing interpolation and orientation conditions directly (interpolation versus orientation and orientation versus interpolation). These first level contrast images were then used to conduct a second level group random effects analysis [84], [85]. Any inferences drawn from the data could therefore be generalised to the population from which the observers were drawn. A threshold of p<0.001, uncorrected for multiple comparisons, was applied to all contrasts, unless otherwise stated.

Whole brain analyses were initially performed using both normalised data (co-registered to the SPM EPI template) and non-normalised data (co-registered to each participant́s T1 anatomical image). As the pattern of results was indistinguishable for normalised and non-normalised data only the former have been presented here. In order to visually explore parameter estimates within activated regions, the MarsBar toolbox (http://marsbar.sourceforge.net/) was used to extract and average parameter estimates for display purposes in activated voxels falling within a sphere (radius 10 mm) centred on peak coordinates of activation identified in the statistical contrasts.

### Eye tracking in the scanner

Eye movements were monitored throughout using an ASL504 LRO infrared video-based MRI compatible eye tracker (Applied Science Laboratory, Bedford, MA) and analysed in the horizontal and vertical plane independently using a custom-written Matlab program. Saccades were defined as any eye movement exceeding a velocity of 30 degrees per second with an amplitude greater than 1 degree of visual angle and less than 20 degrees. In addition, to reduce noise artifacts individual eye movements had to be followed by a fixation period of 50 ms or more to be included in the analysis.

## References

[1] Hubel DH, Wiesel TN (1968). Receptive fields and functional architecture of monkey striate cortex.. J Physiol.

[2] Ringach DL (2004). Mapping receptive fields in primary visual cortex.. J Physiol.

[3] Marr D (1982). Vision. A computational investigation into the human representation and processing of visual information.. Freeman WHC.

[4] Field DJ, Hayes A, Hess RF (1993). Contour integration by the human visual system: evidence for a local “association field”.. Vision Res.

[5] Hess RF, Dakin SC (1997). Absence of contour linking in peripheral vision.. Nature.

[6] Field D, Hayes A, Chalupa LM, JS Wener JS (2001). Contour Integration and the Lateral Connections of V1 Neurons.. The Visual Neurosciences.

[7] Hess R, Field D (1999). Integration of contours: new insights.. Trends Cogn Sci.

[8] Watt RJ (1984). Towards a general theory of the visual acuities for shape and spatial arrangement.. Vision Res.

[9] Morgan MJ, Ward RM, Hole GJ (1990). Evidence for positional coding in hyperacuity.. J Opt Soc Am A.

[10] Toet A, Koenderink JJ (1988). Differential spatial displacement discrimination thresholds for Gabor patches.. Vision Res.

[11] Kooi FL, De Valois RL, Switkes E (1991). Spatial localization across channels.. Vision Res.

[12] Morgan MJ, Regan D (1990). Hyperacuity.. Spatial Vision.

[13] Levi DM, Waugh SJ (1996). Position acuity with opposite-contrast polarity features: evidence for a nonlinear collector mechanism for position acuity?. Vision Res.

[14] Burbeck CA (1987). Position and spatial frequency in large-scale localization judgments.. Vision Res.

[15] Morgan MJ, Regan D (1987). Opponent model for line interval discrimination: interval and vernier performance compared.. Vision Res.

[16] Waugh SJ, Levi DM (1995). Spatial alignment across gaps: contributions of orientation and spatial scale.. J Opt Soc Am A Opt Image Sci Vis.

[17] Haxby JV, Grady CL, Horwitz B, Ungerleider LG, Mishkin M (1991). Dissociation of object and spatial visual processing pathways in human extrastriate cortex.. Proc Natl Acad Sci U S A.

[18] Corbetta M, Miezin FM, Shulman GL, Petersen SE (1993). A PET study of visuospatial attention.. J Neurosci.

[19] Faillenot I, Toni I, Decety J, Gregoire MC, Jeannerod M (1997). Visual pathways for object-oriented action and object recognition: functional anatomy with PET.. Cereb Cortex.

[20] Faillenot I, Decety J, Jeannerod M (1999). Human brain activity related to the perception of spatial features of objects.. Neuroimage.

[21] Pourtois G, Vandermeeren Y, Olivier E, de Gelder B (2001). Event-related TMS over the right posterior parietal cortex induces ipsilateral visuo-spatial interference.. Neuroreport.

[22] Shafritz KM, Gore JC, Marois R (2002). The role of the parietal cortex in visual feature binding.. Proc Natl Acad Sci U S A.

[23] Altmann CF, Bulthoff HH, Kourtzi Z (2003). Perceptual organization of local elements into global shapes in the human visual cortex.. Curr Biol.

[24] Kourtzi Z, Tolias AS, Altmann CF, Augath M, Logothetis NK (2003). Integration of local features into global shapes: monkey and human FMRI studies.. Neuron.

[25] Ostwald D, Lam JM, Li S, Kourtzi Z (2008). Neural coding of global form in the human visual cortex.. J Neurophysiol.

[26] Grill-Spector K, Kushnir T, Edelman S, Itzchak Y, Malach R (1998). Cue-invariant activation in object-related areas of the human occipital lobe.. Neuron.

[27] Mendola JD, Dale AM, Fischl B, Liu AK, Tootell RB (1999). The representation of illusory and real contours in human cortical visual areas revealed by functional magnetic resonance imaging.. J Neurosci.

[28] Murray MM, Wylie GR, Higgins BA, Javitt DC, Schroeder CE (2002). The spatiotemporal dynamics of illusory contour processing: combined high-density electrical mapping, source analysis, and functional magnetic resonance imaging.. J Neurosci.

[29] Stanley DA, Rubin N (2003). fMRI activation in response to illusory contours and salient regions in the human lateral occipital complex.. Neuron.

[30] Hess RF, Hayes A (1994). The coding of spatial position by the human visual system: effects of spatial scale and retinal eccentricity.. Vision Res.

[31] Dupont P, Vogels R, Vandenberghe R, Rosier A, Cornette L (1998). Regions in the human brain activated by simultaneous orientation discrimination: a study with positron emission tomography.. Eur J Neurosci.

[32] Talairach J, Tournoux P (1988). Co-planar stereotaxic atlas of the human brain: 3-Dimensional Proportional System – an approach to cerebral imaging.

[33] Raichle ME, MacLeod AM, Snyder AZ, Powers WJ, Gusnard DA (2001). A default mode of brain function.. Proc Natl Acad Sci U S A.

[34] Laurienti PJ (2004). Deactivations, global signal, and the default mode of brain function.. J Cogn Neurosci.

[35] Raichle ME, Snyder AZ (2007). A default mode of brain function: A brief history of an evolving idea.. Neuroimage.

[36] Grill-Spector K, Kourtzi Z, Kanwisher N (2001). The lateral occipital complex and its role in object recognition.. Vision Res.

[37] Grill-Spector K, Kushnir T, Edelman S, Avidan G, Itzchak Y (1999). Differential processing of objects under various viewing conditions in the human lateral occipital complex.. Neuron.

[38] Dumoulin SO, Bittar RG, Kabani NJ, Baker CL, Le Goualher G (2000). A new anatomical landmark for reliable identification of human area V5/MT: a quantitative analysis of sulcal patterning.. Cereb Cortex.

[39] Paus T (1996). Location and function of the human frontal eye-field: a selective review.. Neuropsychologia.

[40] Mort DJ, Perry RJ, Mannan SK, Hodgson TL, Anderson E (2003). Differential cortical activation during voluntary and reflexive saccades in man.. Neuroimage.

[41] Grosbras MH, Lobel E, Van de Moortele PF, LeBihan D, Berthoz A (1999). An anatomical landmark for the supplementary eye fields in human revealed with functional magnetic resonance imaging.. Cereb Cortex.

[42] Shmuel A, Yacoub E, Pfeuffer J, Van de Moortele PF, Adriany G (2002). Sustained negative BOLD, blood flow and oxygen consumption response and its coupling to the positive response in the human brain.. Neuron.

[43] Wade AR (2002). The negative BOLD signal unmasked.. Neuron.

[44] Amedi A, Malach R, Pascual-Leone A (2005). Negative BOLD differentiates visual imagery and perception.. Neuron.

[45] Laurienti PJ, Burdette JH, Wallace MT, Yen YF, Field AS (2002). Deactivation of sensory-specific cortex by cross-modal stimuli.. J Cogn Neurosci.

[46] Tomasi D, Ernst T, Caparelli EC, Chang L (2006). Common deactivation patterns during working memory and visual attention tasks: an intra-subject fMRI study at 4 Tesla.. Hum Brain Mapp.

[47] Haxby JV, Horwitz B, Ungerleider LG, Maisog JM, Pietrini P (1994). The functional organization of human extrastriate cortex: a PET-rCBF study of selective attention to faces and locations.. J Neurosci.

[48] James TW, Humphrey GK, Gati JS, Menon RS, Goodale MA (2002). Differential effects of viewpoint on object-driven activation in dorsal and ventral streams.. Neuron.

[49] Shikata E, Hamzei F, Glauche V, Knab R, Dettmers C (2001). Surface orientation discrimination activates caudal and anterior intraparietal sulcus in humans: an event-related fMRI study.. J Neurophysiol.

[50] Faillenot I, Sunaert S, Van Hecke P, Orban GA (2001). Orientation discrimination of objects and gratings compared: an fMRI study.. Eur J Neurosci.

[51] Vandenberghe R, Dupont P, De Bruyn B, Bormans G, Michiels J (1996). The influence of stimulus location on the brain activation pattern in detection and orientation discrimination. A PET study of visual attention.. Brain.

[52] Kitada R, Kito T, Saito DN, Kochiyama T, Matsumura M (2006). Multisensory activation of the intraparietal area when classifying grating orientation: a functional magnetic resonance imaging study.. J Neurosci.

[53] Pins D, Meyer ME, Foucher J, Humphreys G, Boucart M (2004). Neural correlates of implicit object identification.. Neuropsychologia.

[54] Orban GA, Dupont P, Vogels R, Bormans G, Mortelmans L (1997). Human brain activity related to orientation discrimination tasks.. Eur J Neurosci.

[55] Navon D (1977). Forest Before Trees: The Precedence of Global Features in Visual Perception Cognitive Psychology.

[56] Fink GR, Halligan PW, Marshall JC, Frith CD, Frackowiak RS (1996). Where in the brain does visual attention select the forest and the trees?. Nature.

[57] Taira M, Tsutsui KI, Jiang M, Yara K, Sakata H (2000). Parietal neurons represent surface orientation from the gradient of binocular disparity.. J Neurophysiol.

[58] Tsutsui K, Jiang M, Yara K, Sakata H, Taira M (2001). Integration of perspective and disparity cues in surface-orientation-selective neurons of area CIP.. J Neurophysiol.

[59] Tsao DY, Vanduffel W, Sasaki Y, Fize D, Knutsen TA (2003). Stereopsis activates V3A and caudal intraparietal areas in macaques and humans.. Neuron.

[60] Sakata H, Taira M, Kusunoki M, Murata A, Tanaka Y (1997). The TINS Lecture. The parietal association cortex in depth perception and visual control of hand action.. Trends Neurosci.

[61] Harris IM, Egan GF, Sonkkila C, Tochon-Danguy HJ, Paxinos G (2000). Selective right parietal lobe activation during mental rotation: a parametric PET study.. Brain.

[62] Harris IM, Miniussi C (2003). Parietal lobe contribution to mental rotation demonstrated with rTMS.. J Cogn Neurosci.

[63] Cant JS, Valyear KF, Goodale MA (2004). ‘Stuff’ versus ‘things’: Neural processing of the material properties and geometric form of objects in human visual pathways.. Journal of Vision.

[64] Rice NJ, Valyear KF, Goodale MA, Milner AD, Culham JC (2007). Orientation sensitivity to graspable objects: an fMRI adaptation study.. Neuroimage.

[65] Harris IM, Benito CT, Ruzzoli M, Miniussi C (2008). Effects of right parietal transcranial magnetic stimulation on object identification and orientation judgments.. J Cogn Neurosci.

[66] Goodale MA, Milner AD, Jakobson LS, Carey DP (1991). A neurological dissociation between perceiving objects and grasping them.. Nature.

[67] Goodale MA, Milner AD (1992). Separate visual pathways for perception and action.. Trends Neurosci.

[68] Goodale MA, Westwood DA (2004). An evolving view of duplex vision: separate but interacting cortical pathways for perception and action.. Curr Opin Neurobiol.

[69] Valyear KF, Culham JC, Sharif N, Westwood D, Goodale MA (2006). A double dissociation between sensitivity to changes in object identity and object orientation in the ventral and dorsal visual streams: a human fMRI study.. Neuropsychologia.

[70] Aso T, Hanakawa T, Matsuo K, Toma K, Shibasaki H (2007). Subregions of human parietal cortex selectively encoding object orientation.. Neurosci Lett.

[71] Altmann CF, Grodd W, Kourtzi Z, Bulthoff HH, Karnath HO (2005). Similar cortical correlates underlie visual object identification and orientation judgment.. Neuropsychologia.

[72] Kellman PJ, Guttman SE, Wickens TD, Kellman TFSaPJ (2001). Models of Segmentation and Grouping.. From Fragments to Objects: Segmentation and Grouping in Vision.

[73] Wertheimer M, Ellis W (1938). Laws of Organization in Perceptual Forms. First published as Untersuchungen zur Lehre von der Gestalt II, in Psycologische Forschung, 4, 301–350.. A source book of Gestalt psychology.

[74] Boucart M, Humphreys GW (1992). Global shape cannot be attended without object identification.. J Exp Psychol Hum Percept Perform.

[75] Boucart M, Humphreys GW (1994). Attention to orientation, size, luminance, and color: attentional failure within the form domain.. J Exp Psychol Hum Percept Perform.

[76] Boucart M, Humphreys GW, Lorenceau J (1995). Automatic access to object identity: attention to global information, not to particular physical dimensions, is important.. J Exp Psychol Hum Percept Perform.

[77] Boucart M, Humphreys GW (1997). Integration of physical and semantic information in object processing.. Perception.

[78] Humphreys GW, Boucart M (1997). Selection by color and form in vision.. J Exp Psychol Hum Percept Perform.

[79] Boucart M, Meyer ME, Pins D, Humphreys GW, Scheiber C (2000). Automatic object identification: an fMRI study.. Neuroreport.

[80] Pins D, Boucart M, Meyer ME, Jack F (2001). Automatic object identification in a perceptual matching paradigm: An fMRI study.. Journal of Vision.

[81] Kourtzi Z, Grill-Spector K, Clifford GRC (2005). fMRI adaptation: a tool for studying visual representations in the primate brain.. Fitting the Mind into the World.

[82] Paus T (2005). Inferring causality in brain images: a perturbation approach.. Philos Trans R Soc Lond B Biol Sci.

[83] Brainard DH (1997). The Psychophysics Toolbox.. Spat Vis.

[84] Friston KJ, Holmes AP, Price CJ, Buchel C, Worsley KJ (1999). Multisubject fMRI studies and conjunction analyses.. Neuroimage.

[85] Friston KJ, Holmes AP, Worsley KJ (1999). How many subjects constitute a study?. Neuroimage.

